# Contrasting population genomic structuring of northern pike ( *Esox lucius* L.) in fresh‐ and brackish water environments: Implications for management and conservation

**DOI:** 10.1111/jfb.70417

**Published:** 2026-03-30

**Authors:** Alfonso Diaz‐Suarez, María‐Eugenia López, Göran Sundblad, Anti Vasemägi

**Affiliations:** ^1^ Chair of Aquaculture, Institute of Veterinary Medicine and Animal Sciences Estonian University of Life Sciences Tartu Estonia; ^2^ Department of Aquatic Resources Institute of Freshwater Research, Swedish University of Agricultural Sciences Drottningholm Sweden

**Keywords:** fresh and brackish environments, northern pike, population structure, RAD‐seq, salinity adaptation

## Abstract

Understanding the factors that shape population genetic structure is crucial for advancing evolutionary studies and developing effective management and conservation strategies. The northern pike (*Esox lucius* L.) is a top teleost predator that inhabits fresh and brackish water environments in the northern hemisphere. Pike populations in the brackish Baltic Sea typically display strong genetic structuring, with coastal sympatric populations that separate during spring for spawning in either shallow, sheltered brackish bays or in freshwater tributaries and wetlands. In contrast to the Baltic Sea, genomic structuring in freshwater environments, particularly in large lacustrine systems, remains poorly understood. To address this gap, we used restriction site‐associated DNA‐sequencing to assess the genetic structure and diversity of northern pike in two ecologically contrasting habitats: freshwater Vänern Lake, Sweden (8932 single nucleotide polimorphisms [SNPs]), and the brackish Baltic Sea around Saaremaa, Estonia (6899 SNPs). The results show strong genetic structuring and lower genetic diversity in brackish environment compared to the higher genetic diversity and extremely low genetic structuring observed in freshwater habitat. We found no evidence of divergent selection within environments. However, we identified 187 outlier SNPs and 62 outlier genes distinguishing the brackish and freshwater environments, potentially reflecting adaptation to salinity. Notably, several of these genes are associated with key biological processes, including osmotic stress regulation (*akap13*), early development (*tfap2a*) and pathogens response (*tlr18*). From a fisheries management perspective, our results indicate that the freshwater system can be managed as a single stock, while strong population structuring among Baltic coastal pike likely requires either large‐scale solutions and/or population‐specific fine‐scale management efforts to maintain the genetic and life‐history diversity among brackish coastal pike populations.

## 1 INTRODUCTION

The sustainable management and conservation of fisheries resources are critical for maintaining the biodiversity, ecosystem health and human livelihoods that depend on aquatic ecosystems (Lemopoulos et al., 2019; Action, 2020). Genetic structuring within fish populations provides key insights into the evolutionary, ecological and demographic processes shaping these resources (Allendorf et al., 2012; Waples & Gaggiotti, 2006). By understanding the genetic connectivity among populations and local adaptations associated with different environmental factors, fisheries managers are able to design strategies that align with the ecology and temporal trends of target species, ensuring their resilience and sustainability. Furthermore, knowledge of genetic structuring is critical for fisheries stock identification and management efforts to preserve genetic diversity, a cornerstone of adaptability and long‐term population viability (Laikre et al., 2010).

Recent advances in genomic technologies have revolutionised our ability to assess and utilise genetic information for fisheries management and conservation (Allendorf et al., 2010; Ouborg et al., 2010; Theissinger et al., 2023). High‐throughput sequencing techniques enable the analysis of entire or substantial genomic regions, providing unprecedented resolution for detecting population structure, adaptive variation and migration patterns (Formenti et al., 2022; Vasemägi et al., 2023). As a result, genome‐wide approaches provide insights that traditional genetic markers may overlook (Lemopoulos et al., 2019; Vasemägi et al., 2023). Thus, population genomics is emerging as a powerful addition to the toolkit for sustainable fisheries management in the face of rapid ecological and climatic changes (Andersson et al., 2024; Ozerov et al., 2013).

Northern pike (*Esox lucius* L.) is a circumpolar top predator fish of increasing interest in ecological and fisheries research (Forsman et al., 2015). In freshwater and coastal ecosystems, pike is important for ecosystem functioning as well as a focal species for recreational fisheries (Arlinghaus et al., 2018; Craig, 2008). The status of pike population varies across the Baltic Sea, showing signs of population decline in the central and southern regions (Olsson et al., 2023), the outer archipelagos along Sweden's east coast (Eriksson et al., 2011; Ljunggren et al., 2010; Olsson, 2019) and some parts of Finland (Lehtonen et al., 2009). These declines have been attributed to multiple factors, such as overharvesting and intense recreational fishing (Bergström et al., 2022; Van Gemert et al., 2022), loss and deterioration of spawning environments (Hansen et al., 2019; Niemi et al., 2023), increased predation by seals and cormorants (Arlinghaus et al., 2021; Bergström et al., 2022), and increased abundance of the mesopredator three‐spined stickleback (*Gasterosteus aculeatus*) which through predator–prey reversal negatively affects early life stages of pike (Donadi et al., 2020; Nilsson et al., 2019). To mitigate the population decline, several conservation initiatives have been implemented, including wetland restoration (Nilsson et al., 2014; Tibblin et al., 2023) and fishing regulations in the form of seasonal spawning closures (Eklöf et al., 2023).

Genetic structuring in wild populations often occurs when dispersal is limited, either due to inherent physical, behavioural or ecological constraints that cause individuals to remain near their natal sites (Gagnon & Angers, 2006; Hemmer‐Hansen et al., 2007), or as a result of natal homing, where individuals return to their birthplace to spawn (Engstedt et al., 2014). In addition, local adaptation can contribute to genetic structuring by causing genetic differences among populations that experience distinct environmental pressures (Wang & Bradburd, 2014). In the Baltic Sea, sympatric coastal pikes separate during spawning, which takes place in either shallow, wave‐sheltered and vegetated bays and inlets (Hansen et al., 2019; Sundblad et al., 2011; Sundblad & Bergström, 2014) or in freshwater streams and wetlands (i.e. anadromous pike; Engstedt et al., 2010; Larsson et al., 2015). Earlier studies on the population structure of Baltic pike, using a limited number of genetic markers, have predominantly focused on genetic relationships across broad geographical scales (100–1000 km), revealing genetically distinct populations that follow an isolation‐by‐distance (IBD) pattern (Bekkevold et al., 2015; Möller et al., 2021; Wennerström et al., 2017). However, factors beyond geographical distance also contribute to genetic differentiation of Baltic pike. For instance, recent research has found significant genetic divergence between ecotypes and spawning grounds separated by as little as 5 km (Diaz‐Suarez et al., 2022; Nordahl et al., 2019; Sunde et al., 2020, 2022). This suggests that genetic structuring in Baltic Sea pike populations is shaped not only by accurate homing but also by ecological and environmental factors. Notably, the prevalence of anadromy and local adaptation to salinity appear to be important for the genetic structuring of Baltic coastal pike (Engstedt et al., 2010, 2014; Rittweg et al., 2024; Roser et al., 2023). With salinities in parts of the Baltic approaching the tolerance limit for pike (Jacobsen et al., 2017; Jørgensen et al., 2010), finding suitable spawning grounds in enclosed bays or freshwater may serve as an important selective force facilitating natal homing and local adaptation. This is supported by studies on adaptive phenotypic divergence of Baltic pike involving various traits, such as salinity and temperature regime, and levels of suspended materials (Sunde et al., 2018, 2019; Tibblin et al., 2015, 2016). Although natal site fidelity and genetic structuring have also been shown in some freshwater systems (Miller et al., 2001), chemical analyses of stable isotopes indicate that natal homing in freshwater systems may not be as strong as anticipated (Oele et al., 2015). Contrasting fresh and brackish water systems may therefore shed light on the population genomic structuring of pike and the role of the environment‐shaping genetic diversity of the species.

Although several genome assemblies for northern pike have been constructed over the years (Johnson et al., 2024; Pan et al., 2021; Rondeau et al., 2014), genome‐wide studies aiming to shed light on genetic diversity and evolutionary processes are still in their early stages. For example, Johnson et al. (2024) recently generated and annotated a chromosome‐level genome assembly for northern pike, comprising 25 chormosom‐length scaffols, with a total length scaffolds, with a total length of 941 Mbp. They also re‐sequenced 50 individuals from across North American, finding a decline in genetic diversity from northwest to east populations, supporting the eastward colonisation of northern pike after the last deglaciation. In the Baltic region, several studies have explored how environmental factors, such as salinity, and geographical distance shape genome‐wide population structure (Lukyanova et al., 2024; Roser et al., 2023; Sunde et al., 2022). Furthermore, analyses combining a subset of highly differentiated single nucleotide polymorphisms (SNPs) with otolith microchemistry data on habitat use and migration behaviour have also identified greater variation in life‐history strategies than the traditional anadromous vs. brackish resident classification (Rittweg et al., 2024). However, the molecular mechanisms of adaptive divergence in pike are still largely unknown and only a few putative outlier SNPs deviating from neutral expectations have been described (Sunde et al., 2022). Identifying selective footprints of divergent selection across genomes in contrasting environments offers a valuable way to estimate the extent of distinct reproductive units and local adaptation in pike.

1. The primary objective of this study was to characterise genome‐wide patterns of genetic diversity, population structure and local adaptation within and between northern pike populations from freshwater (Lake Vänern) and the brackish Baltic Sea (Saaremaa). By simultaneously studying pike in two contrasting ecosystems at similar spatial scales using a RAD‐seq approach, we aimed to address the following questions: (1) How do genetic diversity and population structuring differ between pike populations in freshwater and brackish water environments when analysed at comparable spatial scales? (2) What demographic‐ and habitat‐related factors are most likely associated with the observed patterns of genetic structuring and diversity? (3) At what spatial scales do genome‐wide SNP data reveal putative footprints of divergent selection, reflecting distinct selective pressures acting on these populations? (4) What implications do the observed genomic patterns have for management and conservation of northern pike inhabiting brackish and freshwater environments?

## 2 MATERIALS AND METHODS

### 2.1 Freshwater pike in Lake Vänern

**FIGURE 1:**
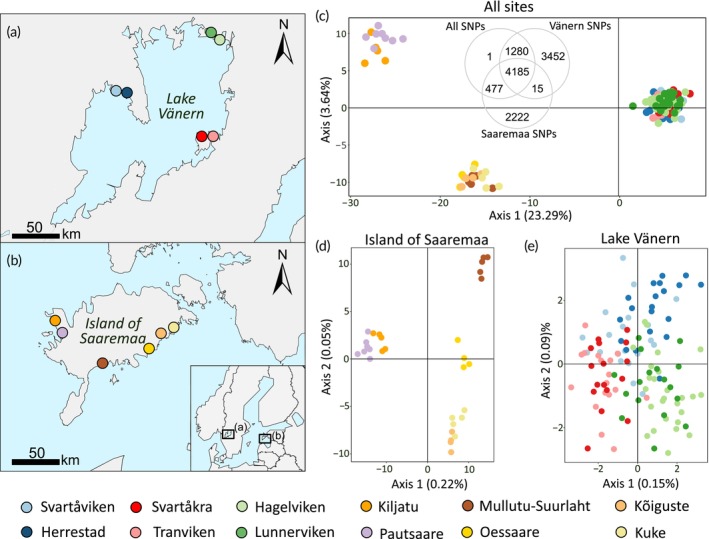
Population structure of northern pike (*Esox lucius* L.) based on single nucleotide polymorphisms (SNP) loci. Sampling sites at (a) Lake Vänern (Sweden) and (b) the island of Saaremaa (Estonia). (c) Discriminant analysis of principal components (DAPC) showing genetic differentiation among all studied individuals based on 5778 SNPs. (d) DAPC of the Baltic pike from Saaremaa based on 6899 SNPs. (e) DAPC of the freshwater pike from Lake Vänern based on 8932 SNPs.

Pike in Lake Vänern were sampled in the spawning season of 2022 (April) during a standardised rod fishing survey in three areas across the lake (Figure 1a). The design and methodology were similar to surveys conducted on the Swedish east coast (see e.g. Eklöf et al., 2023; Ogonowski et al., 2023). In each area, two closely located and similar bays used for spawning were surveyed on two occasions, consisting of 4 days each. Each day, the fishing was divided into a morning and afternoon session of 4 h each. Both bays in an area were fished each day, with alternating morning and afternoon sessions across the 4 days. The first occasion was 1–4 April and the second occasion 23–27 April. Fishing from boats was conducted by two highly experienced anglers with the instruction to catch as many fish as possible. Caught fish were measured for length, weight, spawning status, hooking location and potential injuries, followed by tissue sampling for genetic analysis. Tissue samples were taken as pectoral fin clips preserved in 96% ethanol. After the original survey 49 fin clips had been collected in one area (Karlstad), and 19 and 23 in the other two (Mariestad and Säffle). To reach at least 20 genetic samples per bay, non‐standardised angling was again conducted in May. In total, 129 genetic samples were collected from individuals between 45 and 117 cm (Table 1 and Figure 1a).

**TABLE 1:** Sample information of northern pike (*Esox lucius* L.) from Lake Vänern and the island of Saaremaa.

Lake Vänern	Site	*N*	Average length (cm)	Minimum length (cm)	Maximum length (cm)	Sampling date(s)
Karlstad	Hagelviken	26	75	45	101	01–26.04.2022
	Lunnerviken	23	70	47	117	02–26.04.2022
Mariestad	Svartåkra	20	74	52	115	01.04–30.05.2022
	Tranviken	20	78	52	108	01.04–25.05.2022
Säffle	Herrestad	20	88	60	114	01.04–05.05.2022
	Svartåviken	20	67	51	84	24.04–19.05.2022
Saaremaa						
Northwestern	Kiljatu	5	34	25	43	30.07.2020
	Pautsaare	8	25	5.3	65	06.06.2020
Southeastern	Mullutu‐Suurlaht	5	42	37	49	18.06.2020
	Oessaare	4	26	6.8	32	07.06.2020
	Kõiguste	4	8.5	7.8	10	07.07.2019
	Kuke	5	46.6	42	57.5	17.07.2019

### 2.2 Baltic pike

Pike were collected at six freshwater spawning grounds around the island of Saaremaa (Estonia) during the spring and early summer of 2019 and 2020 (Table 1 and Figure 1b). Fish were captured using standard electrofishing, seine or gillnets, as described in Diaz‐Suarez et al. (2022). The collected individuals included young‐of‐the‐year (YOY) and adults in varying proportions. YOY were euthanized in the field, kept in cold conditions, transported to the laboratory and frozen at −20°C for subsequent analysis. Older individuals were fin clipped in the field and released. In the laboratory, muscle tissue was collected from the YOY. Both tissues were stored in 96% ethanol until subsequent processing.

### 2.3 Ethical Statement

This study complied with all ethical requirements of the *Journal of Fish Biology* national and/or institutional guidelines for the care and use of animals were followed. The fish sampled and handled in this study complied with the standards and procedures stipulated by the Swedish Ministry of Agriculture, and the ethical permit was approved by the Gothenburg ethical committee (DNR 5.8.18‐00761/2021).

### 2.4 Library preparation

DNA from Lake Vänern individuals was isolated from fin clips using a Nucleospin® Tissue kit (Macherey‐Nagel). For Baltic individuals, DNA was extracted from either fin clips or muscle tissue using a DNeasy® blood and tissue kit (Qiagen) (cf. Diaz‐Suarez et al., 2022). DNA concentration was determined using Qubit® dsDNA HS (Thermo Fisher Scientific). A total of 400–600 ng of genomic DNA were digested using EcoRI (Thermo Fisher Scientific). Size selection, library preparation, sequencing (NovaSeq X Plus 25B flowcell 2 × 150 bp), followed by demultiplexing and quality control using MultiQC (Ewels et al., 2016) were performed by SciLifeLab (Sweden). Subsequent SNP calling was carried out using Stacks v2.71 (Catchen et al., 2013) and a northern pike reference genome (NCBI accession: GCA_011004845.1). SNP calling was performed on three different datasets: (i) all individuals, (ii) freshwater individuals from Lake Vänern, and (iii) Baltic individuals (Saaremaa). The SNP loci detected in the three datasets were filtered using dartR v2.9.7 (Gruber et al., 2018) with the following criteria: a minimum call rate of 0.95 per individual, a 0.8 minimum call rate per locus, a minimum allele frequency of 0.05 and exclusion of loci deviating from Hardy–Weinberg equilibrium (*p* < 0.0001). The final SNP dataset included 5778 SNPs across all samples, 8932 SNPs for freshwater pike of Lake Vänern and 6899 SNPs for Baltic individuals. To visualise the level of overlap between the three final SNPs datasets, we constructed a Venn diagram using VennDiagram v1.7.3 (Chen, 2022). The disparity in the number of SNPs obtained after filtering likely emerged from the unequal sample sizes between Vänern (*n* = 121) and Saaremaa (*n* = 31). The 5778 SNP dataset was used to determine general genetic diversity indices, population structure, effective population size (N_e_) and to test for selection signatures for all individuals. The 8932 SNP dataset was used for the population structure analyses of freshwater individuals, and the 6899 SNP dataset was used to perform the population structure analyses of the Baltic individuals.

### 2.5 Genetic diversity, population structure and *N*_e_ estimation

The inbreeding coefficient (*F*_IS_), adjusted observed heterozygosity (*H*_o_) and adjusted (normalised to sample size) expected heterozygosity (*H*_e_) were calculated using dartR v2.9.7 (Gruber et al., 2018). The mean allelic richness was estimated using PopGenReport v3.1 (Adamack & Gruber, 2014; Gruber & Adamack, 2015). The difference in adjusted observed heterozygosity and allelic richness between freshwater and Baltic pike was tested using the nonparametric Mann–Whitney test implemented in stats package v4.1.3 (R Core Team, 2023). To avoid potential bias caused by unequal sample sizes between freshwater and brackish individuals, *H*_o_ (observed heterozygosity) was estimated using the bootstrap approach implemented in poppr R package v2.9.6 (Kamvar et al., 2014), with 1000 replicates and a minimum sample size of four per group. To evaluate the extent to which genetic structuring retains overall genetic diversity (Löytynoja et al., 2023), the same diversity indexes were calculated by separating freshwater and Baltic individuals into two single populations. Genetic differentiation between all sampling sites was determined using a pairwise *F*_ST_ estimator (Weir & Cockerham, 1984). Previous studies have demonstrated that with large SNP panels (≥1500), even small sample sizes (i.e. two individuals per population) can yield reliable *F*_ST_ estimates (Nazareno et al., 2017; Willing et al., 2012). Pairwise *F*_ST_ values, 95% coefficient intervals and significance levels were calculated using stamppFst function in the StAMPP R package v1.6.3 (Pembleton et al., 2013). To explore genetic clustering, we used discriminant analysis of principal components (DAPC) implemented in adegenet v2.1.10 (Jombart, 2008). The optimal number of principal components (PCs) to retain was determined using the a‐score optimization function to avoid underfitting or overfitting the model (Jombart, 2008). To complement DAPC, we inferred population structure using a Bayesian clustering approach implemented in STRUCTURE (Pritchard et al., 2000). To minimise computing time, all STRUCTURE runs were performed using PARALLELSTRUCTURE v1.0, which allows for parallel runs on multi‐core computers (Besnier & Glover, 2013). We conducted three independent runs for each *K* value (*K* = 1 to 12, corresponding to the number of sampling sites), using 100,000 MCMC iterations and a burn‐in of 1000 permutations. The most likely number of clusters (*K*) was determined using Evanno's method (Evanno et al., 2005) for analyses, including all individuals, as well as freshwater and Baltic samples separately. To account the potential effect of linkage disequilibrium (LD) on structure analyses, both DAPC and STRUCTURE were repeated using LD‐filtered SNP datasets. The LD pruning was performed using the gl.filter.ld function from the dartR package (Gruber et al., 2018) applying LD thresholds of 0.5 and 0.2. The procedure identifies and removes loci that exhibit LD greater than the specified cut‐off value.

The effective population size (*N*_e_) was estimated using the LD method implemented in NeEstimator 2.01 (Do et al., 2014). However, *N*_e_ was estimated only in freshwater pike from Lake Vänern because of the small Baltic pike sample size. Finally, the correlation between genetic and waterway distances (isolation‐by‐distance, IBD) for lake and Baltic individuals was determined using the Mantel test implemented in ade4 v1.7 (Dray & Dufour, 2007; Thioulouse et al., 2018). We tested IBD at both individual and population level. At the population level, genetic differences were computed as pairwise *F*_ST_ previously calculated using stamppFst function implemented stamp v1.6.3 (Pembleton et al., 2013). Genetic differences between individuals were estimated as the pair‐wise mean absolute allele frequency differences using poppr v2.9.6 (Kamvar et al., 2014, 2015). Results were visualised using ggplot2 v3.5.1 (Wickham, 2016).

### 2.6 Signatures of selection

Candidate loci under divergent selection were identified using BayeScan v2.1, a method that leverages allele frequency differences between populations using a Bayesian framework (Foll & Gaggiotti, 2008). This approach calculates the posterior probability of loci subjected to selection versus neutrality, with outliers identified as loci showing unusually high divergence relative to neutral expectations. We conducted three different BayeScan runs. To test for signatures of selection in freshwater pike, all Lake Vänern individuals from six locations/populations were used. To identify signatures of selection in Baltic pike, we ran Bayescan using all Saaremaa samples collected from six locations/populations. Finally, we also ran BayeScan for all studied samples consisting of 12 populations, to identify SNPs and genomic regions showing unusually high divergence between freshwater and Baltic Sea environments. All runs were performed using prior odds for the neutral model of 10 (*po* = 10). Loci were identified as candidates under selection if they met the following criteria: a Bayes factor of 3 (−log10 = 0.05), a posterior probability of 0.97 and a positive *α* value, indicating directional selection (Foll & Gaggiotti, 2008).

### 2.7 Gene annotation

To better understand the genomic basis of selection in northern pike genome, all SNPs were annotated to functional categories using SnpEff v4_5 (Cingolani et al., 2012). The SnpEff database was built using the northern pike reference genome (NCBI accession number: GCA_011004845.1). To test for excess and deficiency of identified candidate SNPs in each annotation category, a chi‐squared test was performed using the stats v4.1.3 package (R Core Team, 2023), comparing the frequency of candidate SNPs against all identified SNPs. Finally, Manhattan plots highlighting putative loci under selection were visualised using topr v2.0.2 (Juliusdottir & Stefansson, 2024).

### 2.8 Gene ontology analysis

First, we identified pike genes that are orthologous to human and zebrafish using NCBI datasets v15.29.0. (O'Leary et al., 2024). Subsequently, GO enrichment analysis of the orthologous gene symbols was performed using a binomial test in panther (Ashburner et al., 2000; Aleksander et al., 2023; Thomas et al., 2022).

## 3 RESULTS

### 3.1 Sequencing and genotyping

The initial data set included 3641M raw reads (minimum = 3.6M, maximum = 17.2M, average = 9.8M per individual). In total, 152 individuals were included in the stacks processing. The mean locus coverage across the samples was 61.0x, ranging from 5.8x to 93.0x, with a mean read length of 332.1 bp. The SNPs filtering resulted in 5778 SNPs from all samples. Two individuals did not pass the filtering criteria and were excluded from further analysis, one from Lunnerviken (Vänern Lake) and other from Kiljatu (Saaremaa Island). The dataset comprising only freshwater pike and Baltic individuals consisted of 8932 and 6899 SNPs, respectively. Raw Illumina sequences are available from NCBI under BioProject accession number PRJNA1197401.

### 3.2 Genetic diversity and population structure

Overall genetic diversity estimated as *H*_o_ and *H*_e_ ranged from 0.206 (Kiljatu) to 0.280 (Lunnerviken) and 0.183 (Kiljatu) to 0.273 (Lunnerviken), respectively. Average genetic diversity among freshwater pike populations was higher (*H*_o_ = 0.272, *H*_e_ = 0.269 and AR = 1.684) than in Baltic populations (*H*_o_ = 0.237, *H*_e_ = 0.214 and AR = 1.539), and the differences for all three diversity estimates were significant based on a nonparametric Mann–Whitney test (*p* < 0.05). Similarly, the genetic diversity estimates were higher in the freshwater environment (*H*_o_ = 0.272, AR = 1.97) compared to the Baltic (*H*_o_ = 0.234, AR = 1.890) when all individuals were grouped together. Within Lake Vänern, all sampling sites showed similar genetic diversity estimates (*H*_o_ = 0.268–0.280, *H*_e_ = 0.266–0.273, AR = 1.677–1.169). In contrast, the northwestern Baltic populations showed lower genetic diversity (mean, *H*_o_ = 0.207, *H*_e_ = 1.94, AR = 1.48) compared to the southeastern Baltic populations (mean, *H*_o_ = 0.252, *H*_e_ = 0.223, AR = 1.566). Similar differences in genetic diversity levels were also observed in the *H*_o_ using bootstrapping to equalise sample sizes (Vänern mean, *H*_o_ = 2.99; Saaremaa mean, *H*_o_ = 1.61; Table S1). The estimated *N*_e_ at the freshwater sites showed no apparent geographical pattern and ranged from 154 (95% confidence interval [CI] 151–159) to 653 (95% CI 576–753) per site (Table 2).

**TABLE 2:** Molecular diversity indices and *N*_e_ estimates for the studied populations.

Lake Vänern	AR	*H*_o_ (SD)	*H*_e_ (SD)	*F*_IS_	*N*_e_ (95% CI)
Svartåviken	1.681	0.27 (0.171)	0.267 (0.152)	0.016	550 (507–600)
Herrestad	1.685	0.27 (0.168)	0.269 (0.152)	0.024	326 (310–344)
Svartåkra	1.677	0.268 (0.166)	0.267 (0.151)	0.019	653 (576–753)
Tranviken	1.681	0.273 (0.181)	0.266 (0.156)	0.004	198 (192–204)
Hagelviken	1.689	0.276 (0.166)	0.272 (0.150)	0.005	424 (404–446)
Lunnerviken	1.692	0.280 (0.174)	0.273 (0.151)	0.000	155 (151–159)
Island of Saaremaa					
Kiljatu	1.453	0.206 (0.250)	0.183 (0.197)	‐0.015	–
Pautsaare	1.517	0.208 (0.214)	0.208 (0.208)	0.061	–
Mullutu‐Suurlaht	1.571	0.249 (0.247)	0.226 (0.190)	0.009	–
Oessaare	1.575	0.266 (0.269)	0.226 (0.192)	‐0.031	–
Kõiguste	1.537	0.239 (0.258)	0.211 (0.194)	0.008	–
Kuke	1.581	0.256 (0.256)	0.230 (0.190)	0.001	–

*Note*: *N*_e_ was not estimated in Saaremaa populations due to small sample sizes. *N*_e_ have been rounded to integers.

Abbreviations: AR, allelic richness; CI, confidence interval; *F*_IS_, fixation index; *H*_e_, expected heterozygosity; *H*_o_, observed heterozygosity; *n*, number of samples; *N*_e_, effective population size; SD, standard deviation.

**FIGURE 2:**
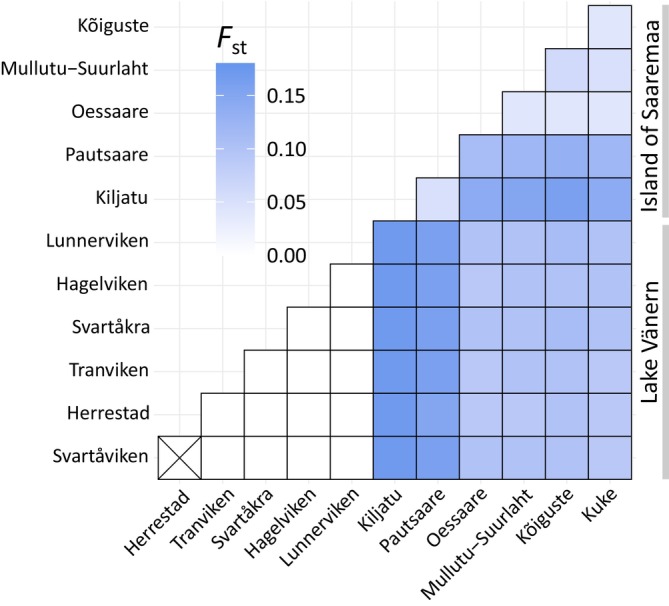
Heatmap of estimated pairwise *F*_ST_ values between studied sites based on 5778 single nucleotide polymorphisms of northern pike (*Esox lucius* L.). The cross symbol indicates non‐significant pairwise differentiation.

Pairwise *F*_ST_ values ranged from 0.009 to 0.172 and were statistically significant in the majority of comparisons (65 out of 66, *p* < 0.05; Figure 2 and Table S2). Freshwater sites showed low genetic differentiation (mean *F*_ST_ = 0.002), with a non‐significant pairwise comparison (Svartåviken vs. Herrestad, *F*_ST_ = 0.0009). In contrast, Baltic populations exhibited a much higher level of genetic divergence, particularly between northwestern and southeastern sites (mean *F*_ST_ = 0.093; Figure 2). The DAPC based on the full dataset clearly separated freshwater and Baltic individuals. Freshwater pike formed a single uniform cluster, while Baltic individuals were divided into two distinct regional clusters: the northwestern and southeastern populations (Figure 1c). The DAPC performed on the Baltic samples alone revealed a more pronounced genetic separation, with the first axis distinguishing northwestern and the southeastern populations, while the second axis further divided southeastern individuals into three distinct clusters (Figure 1d). In contrast, the DAPC of freshwater samples showed weaker structure, forming three partially overlapping clusters corresponding to the bay pairs (Figure 1e). Although DAPC revealed clustering in the freshwater group, the proportion of variance explained by the discriminant axes was low, indicating that the observed groupings likely reflect subtle allele frequency shifts across many loci. This pattern is further accentuated by the high discriminatory power of DAPC, which emphasises differences between groups rather than within them, allowing detection of weak but structured genetic differentiation even under low *F*_ST_ conditions. Similar structuring was obtained with the LD filtered datasets (Figures S1 and S3).

**FIGURE 3:**
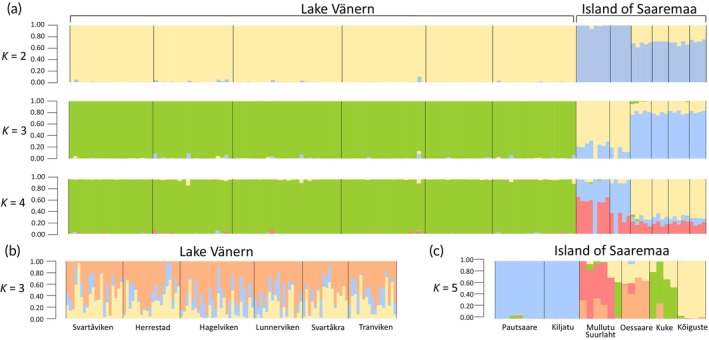
Structure bar plots showing individual membership proportions of northern pike (*Esox lucius* L.) based on 10 runs per *K* value, without prior population information. (a) All individuals (*K* = 2, *K* = 3 and *K* = 4), (b) freshwater pike from Lake Vänern (*K* = 3) and (c) Baltic pike from Island of Saaremaa (*K* = 5). The structure analyses were based on 5778 single nucleotide polymorphisms (SNPs) for all individuals, 8932 SNPs for freshwater pike from Lake Vänern and 6899 SNPs for Baltic pike from Saaremaa.

**FIGURE 4:**
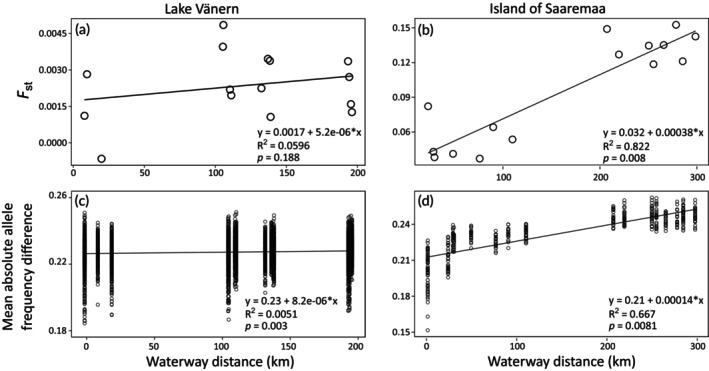
Relationship between genetic differentiation and waterway geographical distance (km) of northern pike (*Esox lucius* L.) between freshwater (Lake Vänern) and Baltic (Saaremaa) populations. Genetic differences were considered at the population level (pairwise *F*_ST_, a and b) and at the individual level (pairwise mean absolute allele frequency differences, c and d).

Structure analysis including all individuals supported *K* = 2 as the most likely number of clusters (based on Evanno's method), separating freshwater and Baltic individuals (Figure 3a and Table S3). However, *K* = 3 also yielded biologically consistent results, distinguishing three main population clusters: freshwater, Baltic northwestern and Baltic southeastern (Figure 3a). When STRUCTURE was run only in the freshwater individuals, the most likely number of clusters was three (*K* = 3) (Table S4) with no clear differentiation among sites (Figure 3b). For the Baltic pike alone, *K* = 5 was best supported (Table S5), grouping all northwestern individuals together and dividing the southeastern group into four different clusters (Figure 3c). In both freshwater and Baltic datasets, genetic structuring within groups was more pronounced in DAPC compared to STRUCTURE. DAPC is a non‐parametric approach that reduces dimensionality with PCA and maximises among‐group variation without assuming Hardy–Weinberg or linkage equilibrium (Jombart et al., 2010). In contrast, STRUCTURE is a model‐based method that relies on these assumptions and can be more sensitive to LD and low genetic differentiation (Falush et al., 2003; Pritchard et al., 2000). As a result, DAPC can sometimes reveal subtle genetic structure more clearly than STRUCTURE. Both LD pruning thresholds (LD < 0.5 and 0.2) produced a highly similar pattern of genetic structuring, indicating that LD had little effect on the inferred differentiation (Figures S2 and S3) The Mantel test considering genetic differences among sites revealed a strong and significant IBD pattern in Baltic pike (*R* ^2^ = 0.82, *p* = 0.008; Figure 4a) but not in freshwater pike (*R* ^2^ = 0.05, *p* = 0.188; Figure 4b). When the Mantel test was based on the genetic differences among individuals, a weak but significant IBD was detected in Lake Vänern pike (*R* ^2^ = 0.005, *p* = 0.003; Figure 4c), whereas Baltic pike exhibited a stronger IBD pattern (*R* ^2^ = 0.667, *p* = 0.0081; Figure 4d).

### 3.3 Signatures of selection

**FIGURE 5:**
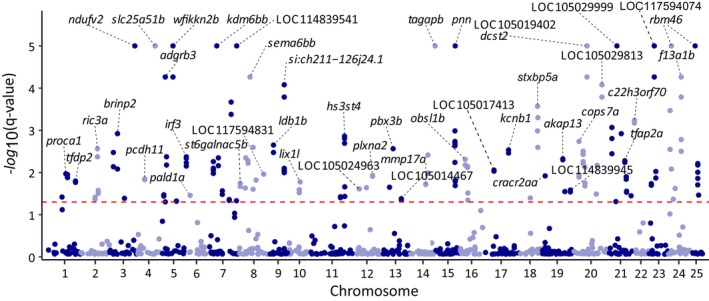
Manhattan plot showing signals of divergent selection between freshwater and brackish environment. The dashed red line indicates a threshold of log_10_(*q*‐value) = 1.32, indicative of strong evidence for selection.

Bayescan analysis of the full dataset (150 individuals across 12 populations) identified 187 SNPs putatively under divergent selection (Figure 5 and Table S7). In contrast, when analysing freshwater and Baltic samples separately, only two and a single outlier locus were observed, respectively. In the full dataset, outlier SNPs were located near or within 62 genes (Supporting Information Table S7). The chi‐squared test detected significant enrichment of candidate loci among the missense variant and splice region variant category (chi‐squared test: *χ* ^2^ = 3.944, *p* = 0.047; Table S6). The highest number of outlier SNPs (five) was observed close to gene *f13a1b* located on chromosome 24, whereas two candidate SNPs were detected at gene *stxbp5a* on chromosome 18 and four SNPs were also detected in unannotated locus (LOC105012317) on chromosome 9 (Figure S4). Among the candidate loci under selection are genes associated with osmotic stress regulation (*akap13*), early development (e.g. *tfap2a*, *nbeaa*, *carmil3*) and response to pathogens (*tlr18* and ifr3). However, the relatively low density of analysed SNPs precluded us from characterising putative footprints of selection and potential causative variants in more detail. Analysis of the gene function showed that outlier genes were not enriched for any biological process, biological component or molecular function terms (FDR ≥ 0.05).

## 4 DISCUSSION

By using genome‐wide markers, we compared the genetic diversity and divergence of pike in two contrasting environments of similar spatial scale: a large freshwater lake and a brackish coastal area of the Baltic Sea. Our findings reveal substantially stronger genetic structuring (mean *F*_ST_ = 0.093) and reduced genetic diversity in the brackish environment, while freshwater populations exhibited higher genetic diversity and minimal genetic structuring (mean *F*_ST_ = 0.002). Furthermore, we found practically no evidence of divergent selection among populations within each environment. However, when considering all samples together, we identified nearly 200 outlier SNPs, suggestive of differences in selective regime between freshwater and brackish water environments. Previous research has revealed pronounced genetic structuring of northern pike populations in the Baltic Sea (Diaz‐Suarez et al., 2022; Möller et al., 2021; Nordahl et al., 2019). In contrast, the genetic structuring and factors driving genetic diversity in large freshwater systems have been explored to a limited extent (Miller et al., 2001; Ouellet‐Cauchon et al., 2014). Below, we examine the factors underlaying contrasting population genetic patterns between the two environments and their implications for managing and preserving the genetic and life‐history diversity of northern pike.

### 4.1 Contrasting population structure and genetic diversity

The divergent genetic patterns observed between Baltic and lake populations raise important questions about the demographic and environment‐related factors driving these differences in genetic structure and diversity. First, it is possible that the reproductive population sizes for pike in Lake Vänern are higher compared to the Baltic pike, making random genetic drift more pronounced in the latter. This is supported by higher levels of genetic diversity in Lake Vänern, reflecting a larger long‐term population size. Furthermore, the estimated effective population sizes for Lake Vänern pike were rather large for a top predator, ranging from 150 to 650 individuals per site. Unfortunately, due to small sample sizes, linkage disequilibrium‐based *N*_e_ estimation was not possible for Baltic pike. We also evaluated if the population substructure of Baltic pike has resulted in preserving genetic diversity at the metapopulation level, as recently shown in lacustrine ringed seal (Löytynoja et al., 2023). However, we did not find evidence that subpopulations had retained enough unique variation that together would result in a similar genetic diversity level as for Lake Vänern.

Second, differences in homing and spawning‐site fidelity between systems may influence the level of gene flow. Baltic pike have shown high spawning‐site fidelity (with 23%–42% returning the following year and none of the fishes being recorded in adjacent spawning habitats, *n* = 1416). They also exhibit short migration distances (normally 3 km, with some fish migrating up to 10 km) and low movement rates between spawning areas (<5%) (Dhellemmes et al., 2023; Engstedt et al., 2014; Flink et al., 2023; Tibblin et al., 2023). Although comparable low movement rates have been observed in freshwater systems (1.3% and 4.8%; Miller et al., 2001), we unfortunately lack information on homing and migration distances in Lake Vänern. However, the observed low genetic divergence and lack of significant IBD pattern suggest that spawning site fidelity in Lake Vänern is likely lower than in the Baltic, contributing to greater gene flow across the lake. Interestingly, based on recreational fishing patterns and anecdotal information from anglers, pike is more frequently caught as a by‐catch in the salmon trolling fisheries in Lake Vänern than in the Baltic, implying that (large) pelagic pikes are more common in the lake. This suggests that increased migration distances associated with pelagic behaviour/ecotype in Vänern may promote broader dispersal and reduced site fidelity. Furthermore, the natural mortality of the pelagic pike is likely lower in freshwater systems, as a continuous decline in the abundance of large Baltic pike has been observed since 1990, coinciding with increasing predation pressure from the Baltic grey seal (*Halichoerus grypus*) (Bergström et al., 2022; Hansson et al., 2018).

Third, we expect the freshwater environment to provide more abundant and continuous spawning habitats than the brackish coastal environment, where spawning is limited to either wetlands and tributaries or shallow, wave‐sheltered bays that warm early in spring (Arlinghaus et al., 2023; Lappalainen et al., 2008; Nilsson et al., 2014). The patchy distribution of spawning habitats in the brackish coastal environment (Sundblad et al., 2011) may therefore promote genetic isolation, as also indicated by the strong IBD pattern observed along the coast. In freshwater habitats, the availability of spawning grounds can also be influenced by water regulation. For example, Ouellet‐Cauchon et al. (2014) showed that large water level fluctuations in Lake St. Pierre reduced pike spawning habitat availability and forced fish to move longer distances to reproduce, ultimately leading to decreased population genetic structure. Although water levels in Lake Vänern are artificially regulated, the current regime is designed to mimic more natural conditions, including higher levels in spring. However, the resulting fluctuations remain relatively small (<0.6 m) and likely have a minor impact on spawning habitat. An additional factor potentially impacting our result is stocking. Although pike are stocked in certain parts of the Baltic, including Estonia (Arlinghaus et al., 2023; Psuty et al., 2023; Wąs‐Barcz et al., 2023), to the best of our knowledge there have been no recent stocking programs in our two study areas prior to sampling.

Fourth, in theory, accurate homing combined with patchy and heterogeneous distribution of suitable spawning environments is expected to facilitate more fine‐scale adaptation to local environmental conditions (Berdahl et al., 2015; Forester et al., 2016). This is further supported by studies on adaptive phenotypic divergence in anadromous pike, which have shown differences in salinity and temperature tolerance, as well as total individual size and growth between spawning grounds (Sunde et al., 2018, 2019; Tibblin et al., 2015). However, our RAD‐seq analyses did not reveal significant adaptive variations within each studied environment. One possible reason for the lack of significant outliers may be the limited number of samples and populations used for analyses (Lotterhos & Whitlock, 2015). This limitation is particularly relevant for our study of Baltic pike, where only a small number of individuals were used. Notwithstanding, we observed a similar lack of outliers when analysing Lake Vänern pike, which have a larger sample size. In this case, the lack of outliers identified may also be related to small number of studied SNPs. Empirical studies with RAD‐seq and whole‐genome sequencing data suggest that both approaches will often recover similar demographic and adaptive signals in a population, although the former is expected to result in footprints of selection at lower resolution (Martchenko & Shafer, 2023). Thus, our genomic analyses suggest that fine‐scale adaptive genetic differences in pike may be less prevalent than previously thought (Berggren et al., 2016), but genome scans at the scale of whole‐genome sequencing are necessary to confirm this.

### 4.2 Adaptive footprints associated with fresh‐ and brackish water environment

We detected 187 outlier SNPs, many of which were associated with 62 annotated genes involved in various cellular and biological processes when both freshwater and Baltic samples were included in the analyses. Several candidate loci under selection have been linked with early development. For example, *hsd17b3* have been linked to ovary development in the olive flounder (*Paralichthys olivaceus*) (Zou et al., 2023) and oocyte proliferation in Siberian sturgeon (*Acipenser baerii*) (Lasalle et al., 2024). In addition, *carmil3* is related to endodermal cell migration during gastrulation (Stark et al., 2022; Stark & Cooper, 2015), *nbeaa* is linked to electrical synaptogenesis (Miller et al., 2015) and *tfap2a* is involved in neural crest induction (Li & Cornell, 2007). Additionally, some outlier SNPs were found in genes related to osmotic stress regulation (*akap13*, Escobar‐Sierra & Lampert, 2024) and response to pathogens (e.g. *tlr18* and *ifr3*; Sun et al., 2010; Trung et al., 2021). On the other hand, none of the 26 outlier genes identified by Sunde et al. (2022) overlapped with our candidate genes. This is not unexpected since Sunde et al. (2022) compared differentiation between anadromous and marine ecotypes, while our study included individuals of lacustrine and anadromous origin. Generally, a low level of parallelism between difference genome scan studies is a rather common phenomenon due to differences is statistical methods and the stochastic nature of the adaptation process (Kess et al., 2024; Perrier et al., 2013; Pettersson et al., 2024). To confirm the presence of adaptive genetic variation associated with brackish and freshwater contrasts, whole‐genome sequencing of larger numbers of populations and individuals will be necessary. Future research should also focus on gaining a deeper understanding of how life‐history variation (anadromy, partial anadromy, sea residency) is associated with genome‐wide differences. This can be achieved by integrating whole‐genome analysis with microchemistry data and conducting common garden experiments to clarify the extent of adaptive divergent selection in pike.

## 5 CONCLUSION AND IMPLICATIONS FOR MANAGEMENT

Genetic structuring and diversity have implications for fisheries management and conservation efforts. The very low genetic structuring combined with lack of evidence for adaptive divergence in the freshwater system suggest that gene flow between subpopulations is sufficient to justify the management of Lake Vänern pike as a single stock (population). In contrast, the fine‐scale structuring observed in Baltic coastal pike requires alternative management approaches if the aim is to maintain genetic diversity and population divergence. Fisheries regulations must either be on spatial scales large enough to include all relevant (sub‐)populations or consist of many fine‐scale measures that target local populations to avoid the overexploitation of less abundant or more vulnerable stocks (Whitlock et al., 2018, 2021). Furthermore, management implementations such as seasonal closures to protect pike spawning populations (Eklöf et al., 2023) or supportive breeding are expected to have different effects in the two ecosystems. For example, site‐specific closures are predicted to benefit the global Vänern population, but not the highly structured populations of Saaremaa Island. Moreover, population supporting actions on Saaremaa Island should include appropriate sources and sufficient number of breeders to maintain genetic diversity and population structure (Cowx, 1994; Epifanio & Waples, 2015; Ryman & Laikre, 1991). Wetland restoration has already been shown to support recruitment of anadromous pike, while also theoretically preserving the genetic diversity and structure of populations (Tibblin et al., 2023). Thus, ongoing wetland restoration activities remain the most suitable management tool for improving the status of coastal Baltic Sea pike. Taken together, our study highlights the fundamental importance and current gaps in knowledge regarding genomic structure, neutral and adaptive variation, and diversity for guiding management and ecological restoration efforts.

## AUTHOR CONTRIBUTIONS

A.V., G.S. and A.D‐S. designed the research. A.D‐S. collected the Estonian samples. G.S. collected the Swedish samples. A.D‐S. generated the data. M.E.L. and A.D‐S. analysed the data. A.V., G.S. and A.D‐S. prepared the manuscript. A.V. and G.S. provided the funding. All authors revised the manuscript and agreed with its publication.

## FUNDING INFORMATION

This work was supported by the Estonian Research Council (grant PRG852 and the project RITA1/02‐60‐05), the Swedish Research Council (grant 2020‐03916) and the Swedish Agency for Marine and Water Management 2024‐001355.

## Supporting information


DATA S1

DATA S2
