# Supplementary material for: Contrasting population genomic structuring of northern pike ( Esox lucius L.) in fresh‐ and brackish water environments: Implications for management and conservation
Source: J Fish Biol. 2026 Mar 30;108(5):1599–613. doi: 10.1111/jfb.70417 (PMC13273091; doi:10.1111/jfb.70417)

**Table 1:** Estimated H_o_ (observed heterozygosity) using bootstrap approach with 1000 replicates and a minimum sample size of four per group

| **Vänern Lake** | **H_o_** |
| --- | --- |
| Svartåviken | 2.99 |
| Herrestad | 2.94 |
| Svartåkra | 2.99 |
| Tranviken | 2.77 |
| Hagelviken | 3.25 |
| Lunnerviken | 2.99 |
| **Island of Saaremaa** |  |
| Kiljatu | 1.60 |
| Pautsaare | 1.60 |
| Mullutu-Suurlaht | 1.60 |
| Oessaare | 1.38 |
| Kõiguste | 1.38 |
| Kuke | 1.61 |

**Table 2:** Genetic differentiation measured using pairwise F*_ST_* based on 5778 SNP loci. Non-significant differentiation (*p* > 0.05) is indicated in bold.

|  | Svartåviken | Herrestad | Tranviken | Svartåkra | Hagelviken | Lunnerviken | Kiljatu | Pautsaare | Oessaare | Mullutu-Suurlaht | Kõiguste |
| --- | --- | --- | --- | --- | --- | --- | --- | --- | --- | --- | --- |
| Svartåviken | - | - | - | - | - | - | - | - | - | - | - |
| Herrestad | **0.0009** | - | - | - | - | - | - | - | - | - | - |
| Tranviken | 0.0012 | 0.0027 | - | - | - | - | - | - | - | - | - |
| Svartåkra | 0.0031 | 0.0039 | 0.0020 | - | - | - | - | - | - | - | - |
| Hagelviken | 0.0019 | 0.0033 | 0.0035 | 0.0047 | - | - | - | - | - | - | - |
| Lunnerviken | 0.0027 | 0.0038 | 0.0022 | 0.0042 | 0.0009 | - | - | - | - | - | - |
| Kiljatu | 0.1684 | 0.1658 | 0.1663 | 0.1724 | 0.1656 | 0.1685 | - | - | - | - | - |
| Pautsaare | 0.1559 | 0.1531 | 0.1557 | 0.1599 | 0.1558 | 0.1560 | 0.0504 | - | - | - | - |
| Oessaare | 0.0958 | 0.0902 | 0.0932 | 0.1000 | 0.0938 | 0.0955 | 0.1372 | 0.1139 | - | - | - |
| Mullutu-Suurlaht | 0.0963 | 0.0923 | 0.0959 | 0.1006 | 0.0970 | 0.0985 | 0.1510 | 0.1239 | 0.0353 | - | - |
| Kõiguste | 0.1049 | 0.1009 | 0.1018 | 0.1121 | 0.1047 | 0.1058 | 0.1576 | 0.1309 | 0.0368 | 0.0623 | - |
| Kuke | 0.0949 | 0.0888 | 0.0941 | 0.0988 | 0.0955 | 0.0992 | 0.1441 | 0.1168 | 0.0404 | 0.0515 | 0.0431 |

**Table 3:** STRUCTURE parameters calculated to detect the number of K using Evanno’s method (Evanno et al., 2005) for all samples. Mean mode of the likelihood distribution (Mean LnP(K)), standard deviation of the mean mode of the likelihood distribution (Stdev LnP(K)), rate of change of the likelihood distribution (Ln´(K)) and absolute values of the second order rate of change of the likelihood distribution (|Ln´´(K)|) and second order rate of change of the likelihood function with respect to K (ΔK).

| K | Reps | Mean LnP(K) | Stdev LnP(K) | Ln'(K) | \|Ln''(K)\| | ΔK |
| --- | --- | --- | --- | --- | --- | --- |
| 1 | 3 | -788185 | 18.9153 | NA | NA | 0 |
| 2 | 3 | -760092 | 33.1919 | 28093.13 | 1698299 | 51166.03 |
| 3 | 3 | -2430298 | 1490424 | -1670206 | 2377087 | 1.594906 |
| 4 | 3 | -1723417 | 947626 | 706881.1 | 2890370 | 3.050117 |
| 5 | 3 | -3906906 | 1085965 | -2183489 | 3267936 | 3.009247 |
| 6 | 3 | -2822458 | 1234037 | 1084447 | 3731628 | 3.023919 |
| 7 | 3 | -5469639 | 2100290 | -2647181 | 1413832 | 0.67316 |
| 8 | 3 | -6702988 | 2947002 | -1233349 | 500508.6 | 0.169837 |
| 9 | 3 | -8436845 | 5148032 | -1733857 | 316525.9 | 0.061485 |
| 10 | 3 | -1E+07 | 2123339 | -2050383 | 6441523 | 3.033676 |
| 11 | 3 | -6096088 | 2269609 | 4391140 | 6360820 | 2.802606 |
| 12 | 3 | -8065768 | 2920031 | -1969680 | NA | NA |

**Table 4:** STRUCTURE parameters calculated to detect the number of K using Evanno’s method (Evanno et al., 2005) for Lake Vänern samples. Mean mode of the likelihood distribution (Mean LnP(K)), standard deviation of the mean mode of the likelihood distribution (Stdev LnP(K)), rate of change of the likelihood distribution (Ln´(K)) and absolute values of the second order rate of change of the likelihood distribution (|Ln´´(K)|) and second order rate of change of the likelihood function with respect to K (ΔK).

| K | Reps | Mean LnP(K) | Stdev LnP(K) | Ln'(K) | \|Ln''(K)\| | ΔK |
| --- | --- | --- | --- | --- | --- | --- |
| 1 | 3 | -920168 | 62.044 | NA | NA | NA |
| 2 | 3 | -921385 | 1678.754 | -1217.2 | 49738.47 | 29.6282 |
| 3 | 3 | -972341 | 7768.382 | -50955.7 | 411845.1 | 53.01556 |
| 4 | 3 | -1435142 | 38145.72 | -462801 | 164008.7 | 4.299532 |
| 5 | 3 | -2061951 | 237665.4 | -626810 | 988261.1 | 4.158203 |
| 6 | 3 | -3677022 | 725754.7 | -1615071 | NA | NA |

**Table 5:** STRUCTURE parameters calculated to detect the number of K using Evanno’s method (Evanno et al., 2005) for island of Saaremaa samples. Mean mode of the likelihood distribution (Mean LnP(K)), standard deviation of the mean mode of the likelihood distribution (Stdev LnP(K)), rate of change of the likelihood distribution (Ln´(K)) and absolute values of the second order rate of change of the likelihood distribution (|Ln´´(K)|) and second order rate of change of the likelihood function with respect to K (ΔK).

| K | Reps | Mean LnP(K) | Stdev LnP(K) | Ln'(K) | \|Ln''(K)\| | ΔK |
| --- | --- | --- | --- | --- | --- | --- |
| 1 | 3 | -181896 | 39.0704 | NA | NA | NA |
| 2 | 3 | -176968 | 3115.591 | 4928.5 | 212560.1 | 68.22467 |
| 3 | 3 | -384599 | 102625 | -207632 | 40945.23 | 0.398979 |
| 4 | 3 | -551286 | 288816.2 | -166686 | 173320.8 | 0.600107 |
| 5 | 3 | -891293 | 170067 | -340007 | 527893.7 | 3.104033 |
| 6 | 3 | -703406 | 352842.7 | 187886.5 | NA | NA |

Reference

Evanno, G., Regnaut, S., & Goudet, J. (2005). Detecting the number of clusters of individuals using the software STRUCTURE: A simulation study. Molecular Ecology, 14(8), 2611–2620. https://doi.org/10.1111/j.1365-294X.2005.02553.x

**Table 6**: Enrichment and depletion of candidate SNPs in each annotation category.

| Variant | All SNP | All SNP (%) | Candidate SNP | Candidate SNP (%) | chi-squared (x^2^) | P value |
| --- | --- | --- | --- | --- | --- | --- |
| 5’ UTR variant | 66 | 0.547 | 3 | 0.857 | 0.133 | 0.715 |
| Downstream gene variant | 1343 | 11.130 | 32 | 9.143 | 1.183 | 0.277 |
| Intergenic region | 2612 | 21.646 | 88 | 25.143 | 0.979 | 0.322 |
| Intragenic variant | 117 | 0.970 | 7 | 2.000 | 2.416 | 0.120 |
| Intron variant | 2971 | 24.621 | 92 | 26.286 | 0.092 | 0.762 |
| Missense variant | 53 | 0.439 | 1 | 0.286 | 0.002 | 0.966 |
| Missense variant and splice region variant | 10 | 0.083 | 2 | 0.571 | 3.944 | 0.047 |
| Non coding transcript exon variant | 25 | 0.207 | 2 | 0.571 | 0.685 | 0.408 |
| Non coding transcript variant | 3241 | 26.858 | 93 | 26.571 | 0.049 | 0.825 |

**
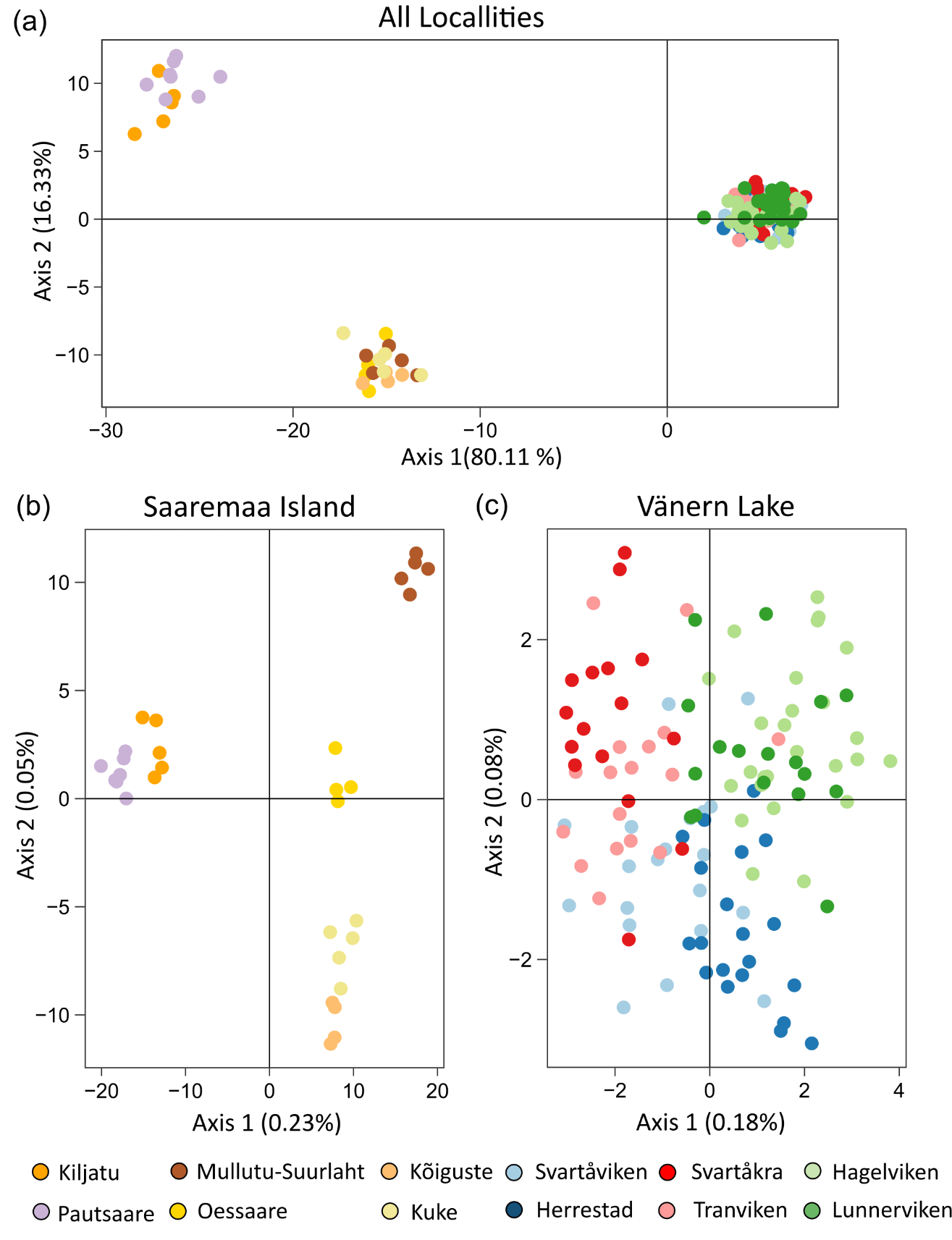
Figure 1:** Population structure of northern pike (*Esox lucius* L.) based on LD < 0.5 threshold. (a) Discriminant Analysis of Principal Components (DAPC) of genetic differentiation for all studied individuals based in 5529 SNPs. (b) DAPC of the Baltic pike from Saaremaa based on 5820 SNPs. (c) DAPC of the freshwater pike from Lake Vänern based on 7329 SNPs.


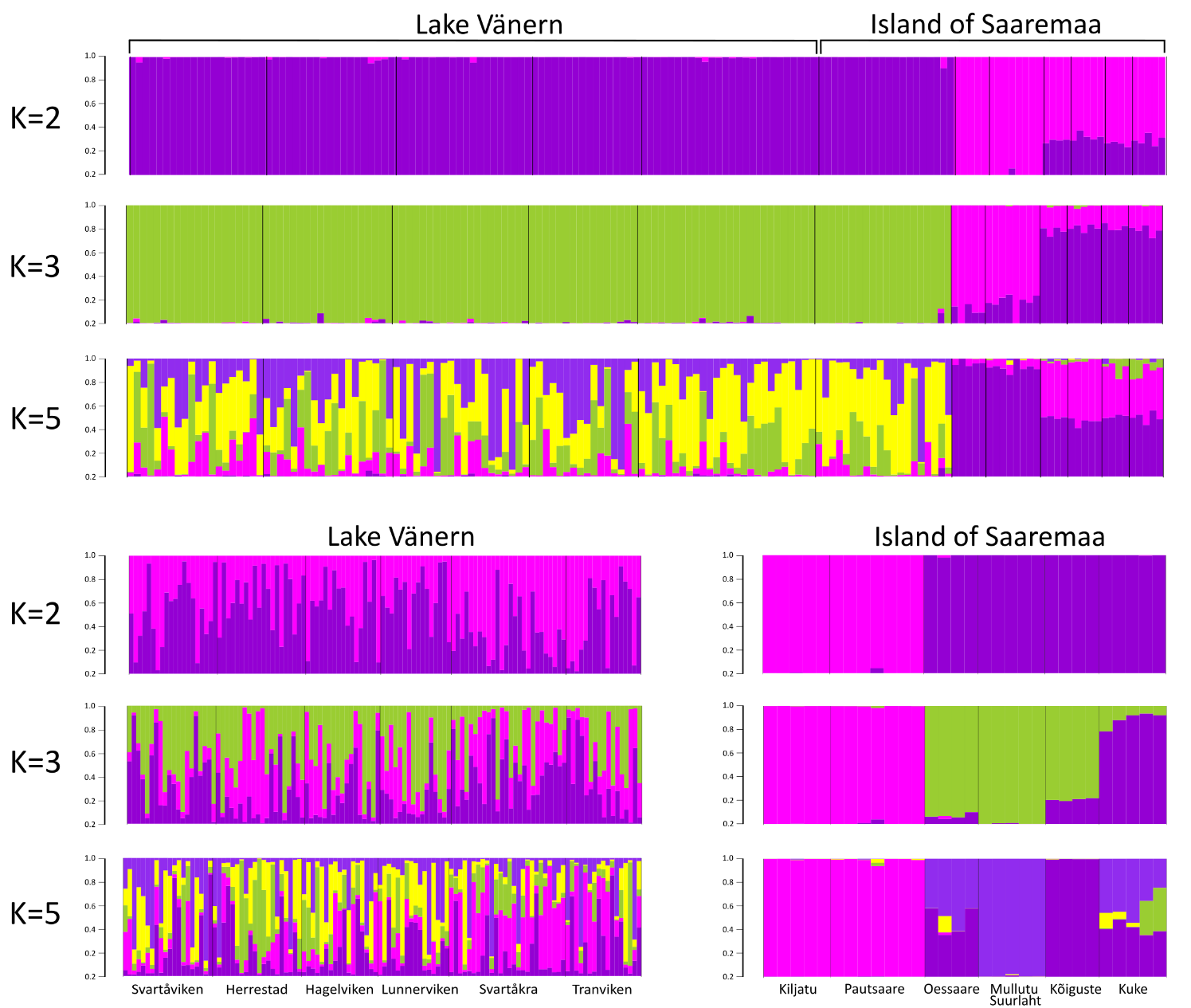


**Figure 2**: Individual population membership of northern pike (*Esox lucius* L.) based on LD < 0.5 threshold, no prior population information and 10 runs per K using STRUCTURE. The plots show the three K optimal values for each dataset: all individuals (K = 2, upper), freshwater pike from Lake Vänern (K = 3, middle), and Baltic pike from Island of Saaremaa (K = 5, bottom). The structure analysis is based LD filtered datasets including 5529 SNPs for all individuals, 7329 SNPs for freshwater pike from Lake Vänern, and 5820 SNPs for Baltic pike from Saaremaa.


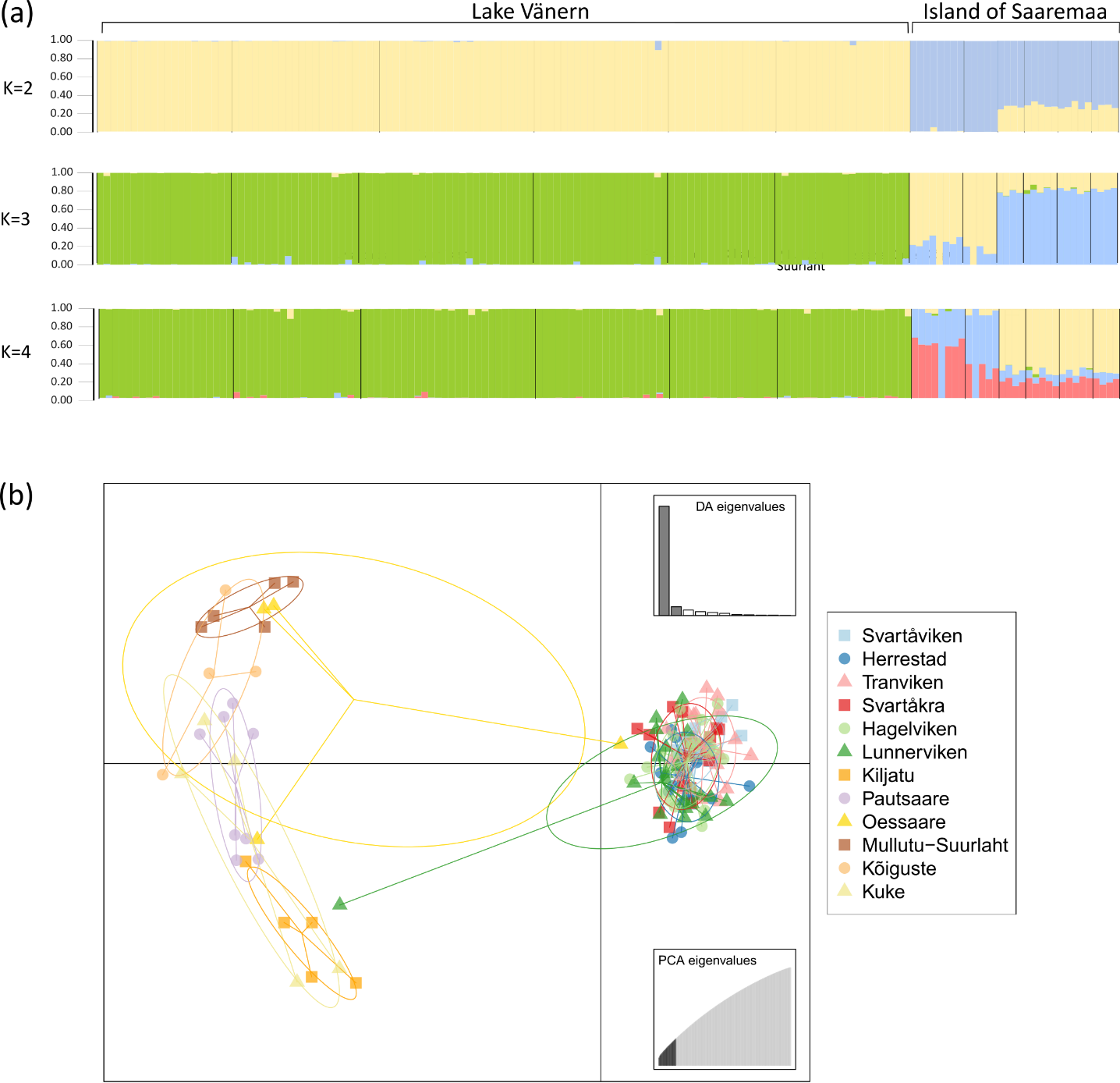


**Figure 3**: Population structure of northern pike (*Esox lucius* L.) based on LD < 0.2 threshold. (a) Discriminant Analysis of Principal Components (DAPC) of genetic differentiation for all studied individuals based in 2347 SNPs. (b) Individual population membership of Northern pike (*Esox lucius* L.) based on no prior population information and 10 runs per K using STRUCTURE including 2347 SNPs.

**Figure 4:** Manhattan plots of genomic regions with highly divergent loci and nearby genes. Gene symbols are according to the northern pike (*Esox lucius* L.) reference genome (GCA_011004845.1). Protein coding (green), lncRNA (red)
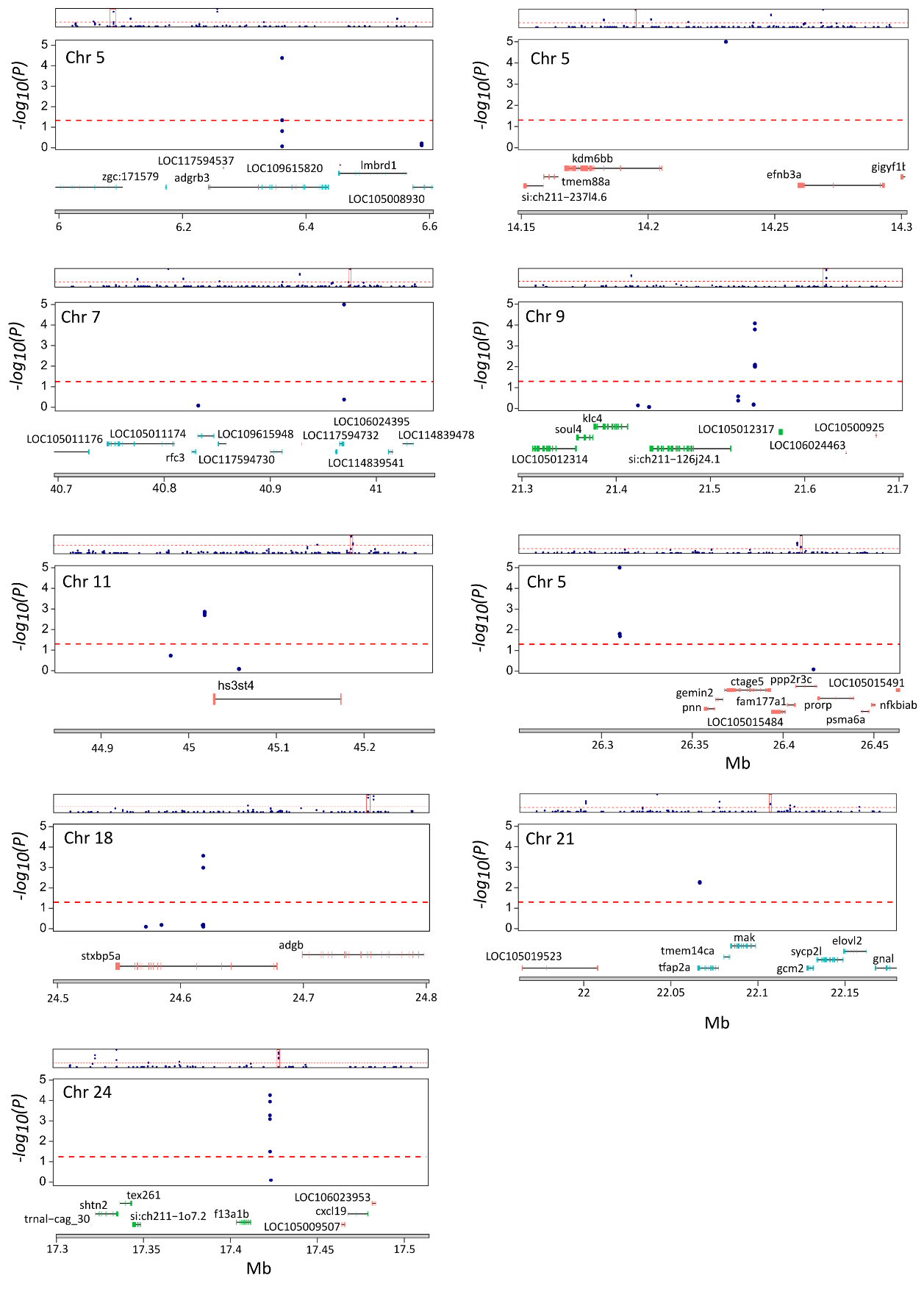

Supplement: Supplementary file 2 — DATA S2 [file JFB-108-1599-s002.docx]
